# The expression profile of microRNAs in a model of 7,12-dimethyl-benz[*a*]anthrance-induced oral carcinogenesis in Syrian hamster

**DOI:** 10.1186/1756-9966-28-64

**Published:** 2009-05-13

**Authors:** Tao Yu, Xiao-yi Wang, Ren-guo Gong, An Li, Sen Yang, Yu-tang Cao, Yu-ming Wen, Chang-mei Wang, Xin-zhu Yi

**Affiliations:** 1 Department of Oral Maxillofacial Surgery, West China College of Stomatology, Sichuan University, No.14, Sec.3, Renminnan Road, Chengdu, Sichuan 610041, PR China; 2 Department of Stomatology, Affiliated Hospital of Panzhihua College, No.76, Jian She Street, Panzhihua, Sichuan 617000, PR China; 3 Department of Stomatology, Affiliated Shougang Hospital of Peking University, Xi Huang Village, Shi Jing Shan District, Peking 100041, PR China; 4 Department of Temporomandibular Joint, West China College of Stomatology, Sichuan University, No.14, Sec.3, Renminnan Road, Chengdu, Sichuan 610041, PR China; 5 State Key Laboratory of Oral Diseases, Sichuan University, No.14, Sec.3, Renminnan Road, Chengdu, Sichuan 610041, PR China

## Abstract

**Background:**

Non-coding RNA molecules, such as microRNAs, may play an important role in carcinogenesis. Recent studies have indicated that microRNAs are involved in initiation and progression of various malignancies. However, little work has been done to compare the microRNA expression patterns in oral cancer. In this study, we constructed an animal model of oral squamous cell carcinoma to investigate expression profiles of microRNAs in oral carcinogenesis.

**Methods:**

The animal model of oral squamous cell carcinoma was conducted by tri-weekly (Monday, Wednesday, and Friday) painting with 5% DMBA in acetone. Six Syrian hamsters, including three from the treated group and three from the control group, were used as a training group for microRNA microarray analysis. All microarray data were analyzed by Significance Analysis of Microarrays (SAM) and CLUSTER 3.0 software, and this result was further confirmed by qRT-PCR assay.

**Results:**

Seventeen microRNAs were differentially expressed in oral squamous cell carcinoma. Five microRNAs (hsa-miR-21, hsa-miR-200b, hsa-miR-221, hsa-miR-338, and mmu-miR-762) were significantly upregulated and twelve microRNAs (hsa-miR-16, hsa-miR-26a, hsa-miR-29a, hsa-miR-124a, hsa-miR-125b, mmu-miR-126-5p, hsa-miR-143, hsa-miR-145, hsa-miR-148b, hsa-miR-155, hsa-miR-199a, and hsa-miR-203) were down-regulated in cancer tissues. The expression levels of hsa-miR-21 and hsa-miR-16 seen with Stem-loop qRT-PCR were also seen in microarray analysis in all samples.

**Conclusion:**

Our findings identified specific microRNA expression in oral squamous cell carcinoma and suggested that microRNAs have a role in oral carcinogenesis.

## Background

MicroRNAs (miRNAs) are small, noncoding RNAs (~20–22 nucleotides) that have critical functions in various biological processes [1]. These naturally occurring miRNAs function by binding to target mRNAs, resulting in the degradation or translational inhibition of the mRNA, based upon the degree of complementarity with it. First described in 1993 in the nematode *Caenorhabditis elegans *[2], to date, thousands of miRNAs have been cloned in higher eukaryotes and a number have been shown to play a role in cell proliferation, apoptosis, growth and morphogenesis [3-5]. At present, dysregulation of miRNAs has been shown to be involved in tumor initiation and progression.

The explosion of data on miRNAs and cancer has put them in the spotlight over the past few years. Numerous studies have highlighted the suspected role of miRNAs in tumorigenesis and have established that profiling of these miRNAs represents an informative method for determining developmental lineage and the differentiation state of various malignancies. The initial connection of miRNAs and cancer was elucidated in leukemia and hematological malignancies, later spurring interest in solid malignancies. For example, one of the first lines of evidence for direct involvement of miRNAs in cancer was the finding that miR-15 and miR-16 are located within a 30 kb deletion in chronic lymphocytic leukemia (CLL), and that both genes were deleted or underexpressed in most cases of this cancer [6]. Abnormal expression of microRNAs has been found in a variety of solid tumors, including colon, breast, lung, thyroid, glioblastomas, prostate, lymphomas, ovarian, hepatocellular, cervical, and pancreatic carcinomas [7-17].

Comparatively, oral cancer has received very little attention in this area of genome profiling. It is identified as a significant public health threat worldwide because its treatment often produces dysfunction and distortions in speech, mastication and swallowing, dental health, and even in the ability to interact socially. It is one of the 10 most frequent cancers in human males worldwide, with about two thirds of all cases occurring in developing countries [18]. The most common type of oral cancer is squamous cell carcinoma. At present, the management of oral squamous cell carcinoma (OSCC) includes combinations of surgery, radiotherapy, and chemotherapy [19]. Despite improvements in these therapies, the 5-year survival rate has not improved significantly and remains at about 50% [20]. In clinical practice, treatment planning and prognosis for patients with OSCC are mainly based on the TNM classification. TNM classification provides significant diagnostic information concerning the tumor, but does not give the clinician sufficient therapeutic biological information, such as the metastatic potential or the sensitivity or resistance of the tumor to radiotherapy and chemotherapy [21]. There is an urgent need for diagnostic methods for distinguishing high-risk patients from other patients in order that optimal managements can be applied.

As such, some of the urgent priorities in this field are the need to identify and elucidate novel genes or pathways that may choreograph this disease. In the present study, by using the miRNA microarray technique, we have measured the relative expression of microRNAs in 7,12-dimethyl-benz- [*a*]-anthrance (DMBA)-induced OSCC in Syrian hamster. We hope that it can contribute to early diagnosis and treatment of this malignancy.

## Methods

### Animals

The construction of the animal model was conducted at West China College of Stomatology, Sichuan University. Twenty-four adult male (150 to 250 g) Syrian hamsters (6 weeks old; sydw, Sichuan, China) were randomly divided into two experimental groups (Group A and B) and one control group (Group C) (n = 8 for each group). After one week of acclimatization, both cheek pouches of each animal in the experimental groups were treated with 5% DMBA (Sigma, St Louis, MO, USA) in acetone. DMBA was applied tri-weekly (Monday, Wednesday and Friday) with a paintbrush. The animals from group A received carcinogen for about 12 weeks. Group B received carcinogen about 12 weeks, with an additional 6-week period of observation. Group C received no treatment and served as blank control. The animal groupings and protocol of carcinogen application are summarized in Table 1.

**Table 1:** Protocol and effect of DMBA-induced oral carcinogenesis on cheek pouch of syrian hamster

Group	Animals	Treatment protocol	Histological type				Mean diameter of tumors
			NM	PP	CIS	SCC	(mm)
Experiment Group							
A	7	5%DMBA-12 week-killed	0	1	1	5	5 ± 1.69
B	7	5%DMBA-18 week-killed	0	0	0	7	8.7 ± 2.55
Control Group							
C	8	No treatment-18 week-killed	8	0	0	0	-

5%DMBA:7,12-dimethyl benz [*a*]anthrance in acetone;

NM:normal;PP:papilloma;CIS:carcinoma in situ;SCC:squamous cell carcinoma

All tissue samples were collected under pentobarbital anesthesia for histopathological examination and miRNA microarray. Six Syrian hamsters, including three from group A and B (12 wk, 18 wk, and 18 wk, respectively) and three from group C (blank control group), were used as a training group for miRNA microarray analysis. All of the handling measures used with the Syrian hamsters were in accordance with approved guidelines (Guidelines for the Care and Use of Laboratory Animals) established by the Chinese Council on Animal Care.

### Fabrication of the miRNA microarray

The miRNA microarrays were obtained from CapitalBio Corporation (Beijing, China), corresponding to the current release of the Sanger miRNA database (; August 2007). The individual oligonucleotide probe was printed in triplicate on chemically modified glass slides in a 21 × 21 spot configuration of each subarray. The spot diameter was 130 mm, and distance from center to center was 185 mm. A total of 924 mature miRNA sequences were assembled and integrated into our miRNA microarray design. These microarray probes included 677 human miRNAs (including 122 predicted miRNA sequences) [22], 292 rat, and 461 mouse mature miRNAs from the miRNA Registry. All of the oligonucleotide probes were presented in triplicate in one microarray, and each of the four subarrays contained 16 controls (Zip5, Zip13, Zip15, Zip21, Zip23, Zip25, Y2, Y3, U6, New-U2-R, tRNA-R, hsa-let-7a, hsa-let-7b, hsa-let-7c, 50%DMSO (Dimethyl Sulfoxide), and Hex). The limited sequence length of miRNAs left little consideration for probe design strategy, so all miRNA probe sequences were designed to be complementary to the full-length mature miRNA.

### Nucleic acid extraction, labeling, and hybridization

Total RNA from each tissue sample was extracted with Trizol reagent (Invitrogen, Carlsbad, USA), and the low-molecular-weight RNA was isolated by a PEG solution precipitation method, according to a previous protocol [23]. We adopted the T4 RNA ligase labeling method according to Thomson' protocol; that is, 4 μg of low-molecular-weight RNA was labeled with 500 ng of 5'-phosphate-cytidyl-uridyl-cy3-3' (Dharmacon, Chicago, USA) with 2 units of T4 RNA ligase (NEB, Beijing, China) [24]. The hybridization chamber was laid on a three-phase tiling agitator BioMixerTM II (CapitalBio, Beijing, China) to promote microfluidic circulation under the coverslip. The hybridization was performed in a water bath at 42°C overnight. The array was then washed with two consecutive washing solutions (0.2% SDS, 2 × SSC at 42°C for 5 min, and 0.2% SSC for 5 min at room temperature). This procedure was repeated twice for each sample.

### Microarray imaging and data analysis

The miRNA microarray from CapitalBio Corporation was a single-channel fluorescence chip; all oligonucleotide probes were labeled with Cy3 fluorescent dye (green). Fluorescence scanning used a double-channel laser scanner (LuxScan 10 K/A, CapitalBio). The figure signal was transformed to a digital signal using image analysis software (LuxScan3.0, CapitalBio). Signal intensities for each spot were calculated by subtracting local background from total intensities. Raw data were normalized and analyzed using the Significance Analysis of Microarrays (SAM, version 2.1, Stanford University, CA, USA) software [25]. The raw data was Log2 transformed and median centered by arrays and genes using the adjust data function of CLUSTER 3.0 software for cluster analysis [26].

### Stem-loop qRT-PCR for miRNAs

All miRNA-specific primers were designed according to miRNA sequences. The universally expressed U6 was used as an internal control. Reverse transcriptase reactions contained 2.5 ng/μL purified total RNA, 50 nM stem-loop reverse transcription (RT) primer, 1 × RT buffer, 0.25 mM of each of dNTPs, 3.33 U/ml MultiScribe reverse transcriptase, 0.25 U/ml RNase inhibitor. The 7.5 μL reactions were incubated in an MJ Research PTC-225 Thermocycler for 30 min at 16°C, 30 min at 42°C, 5 min at 85°C, and then held at 4°C. All reverse transcriptase reactions were run in duplicate. Stem-loop qRT-PCR was performed as described in published references [27]. The 10 μl PCR reaction contained 0.67 μl RT product, 1 × PCR Master Mix, 1.5 μM forward primer, and 0.7 μM reverse primer. The reactions were incubated at 95°C for 10 min, followed by 40 cycles of 95°C for 15 s and 60°C for 1 min. All reactions were run in triplicate. Melting curves were performed using Dissociation Curves software (Funglyn) to ensure only a single product was amplified, and the specificity of samples was confirmed by running on a 3% agarose gel. All reagents from MBI Company (MBI Fermentas, Maryland, USA) were used following the manufacturer's protocols.

## Results

### The effect of DMBA-induced oral carcinogenesis

**Figure 1:**
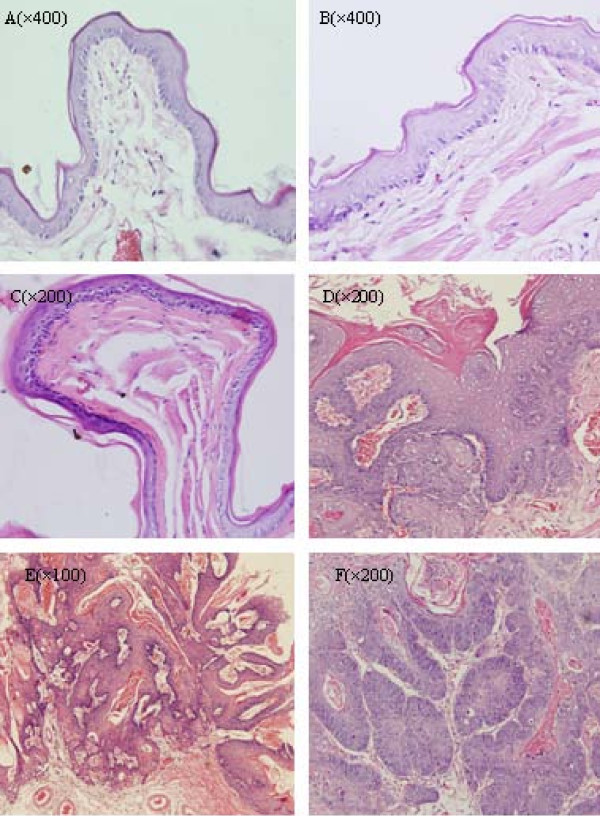
**DMBA-induced oral carcinogenesis in the hamster cheek pouch (H&E staining)**. (A~C) Normal epithelium; (D) SCC; (E) Papillary SCC; (F) SCC.

Two animals died during the experimental period (one each from Groups A and B). Histologically, all samples from Group C appeared normal, with a thin epithelium devoid of rete ridges (Figure 1A~C). Five animals from Group A and seven animals from Group B developed SCC (Figure 1D~F). The tumor diameters ranged from 1.5 mm to 15 mm in both groups, with an average diameter of 5 ± 1.69 mm and 8.7 ± 2.55 mm for Group A and B, respectively (Table 1). Most of the squamous cell carcinomas were classified as well-differentiated or moderately differentiated.

### miRNA microarray analysis

**Figure 2:**
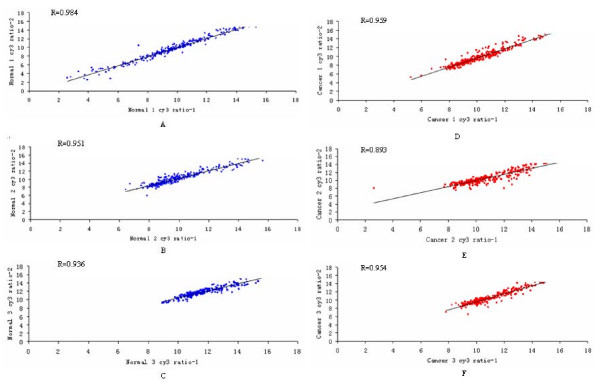
**Experimental variation and reproducibility assessment from twelve microarray hybridizations in six different samples**. Scatter diagram showing high reproducibility between the replicate experiments of every sample. The R-value in each microarray analysis showing that most of the average correlations are well above 0.9, indicating high reproducibility. Panel A~C: self-hybridization results obtained after probing the microarray with the same RNA sample prepared from three normal tissues and labeled separately with Cy3 dye. Panel D~F: self-hybridization results obtained after probing the microarray with the same RNA sample prepared from three cancer tissues and labeled separately with Cy3 dye.

**Figure 3:**
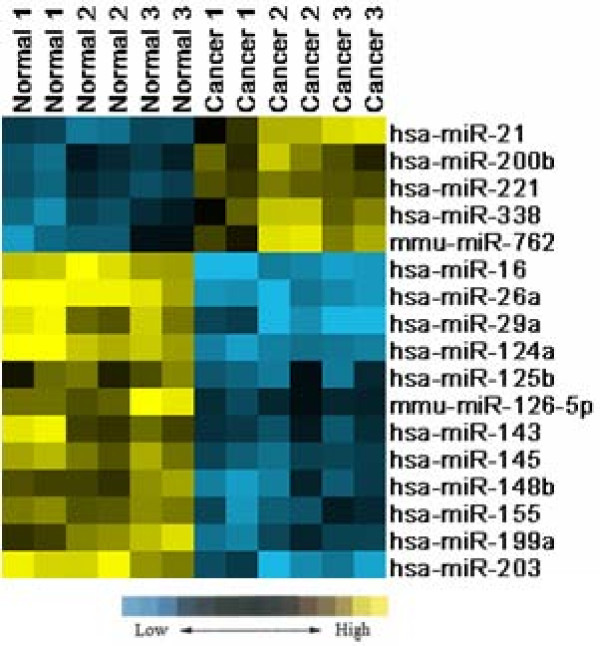
**Supervised hierarchical clustering analysis of miRNA expression**. 17 miRNAs expression profile (from SAM result) of 6 samples were clustered using Cluster 3.0. 6 samples were successfully separated into 2 discrete groups.

RNA gel electrophoresis demonstrated that the quality of the RNA was good. SAM was performed to identify differences in miRNA expression between cancerous and normal samples. SAM calculated a score for each gene on the basis of the change in expression relative to the S.D. of all measurements. The SAM data indicated that 5 miRNA genes were significantly overexpressed and that 12 miRNA genes were significantly underexpressed in cancer samples, with fold changes>2. These results are shown in Table 2. For increased confidence, we repeated each microarray assay twice. The scatter diagrams and correlation assessment of all spots showed that the reproducibility and reliability were good (Figure 2). The supervised cluster analysis, based on differentially expressed miRNAs, generated a tree with clear distinction between cancerous and normal tissues (Figure 3).

**Table 2:** MicroRNAs microarray SAM results and correlation with cancer

microRNA Name	Fold Change	Type	Numerator (r)	Denominator (s+s0)	Correlation with cancer
squamous cell carcinoma vs normal cheek pouch tissue					

hsa-miR-21	2.590	up	2.495	1.371	Up-regulated in glioblastomas[11], breast[8], colon[7], lung[9], pancreatic[17], thyroid[10], and ovarian cancer[15]
hsa-miR-200b	2.192	up	1.645	0.964	Up-regulated in ovarian cancer[15]
hsa-miR-221	2.018	up	1.561	0.988	Up-regulated in CLL[8], glioblastomas[11], thyroid[10], and pancreatic cancer[17]
hsa-miR-338	2.436	up	1.323	0.493	
mmu-miR-762	2.379	up	1.863	1.052	
hsa-miR-16	0.182	down	-2.501	0.458	Down-regulated in CLL[8], and prostate cancer[12]
hsa-miR-26a	0.135	down	-2.288	1.148	Down-regulated in prostate[12], and ovarian cancer[15]
hsa-miR-29a	0.245	down	-1.532	0.785	Down-regulated in ovarian cancer[15]
hsa-miR-124a	0.216	down	-1.819	0.702	Down-regulated in colon[7], breast[8] and lung cancer[9]
hsa-miR-125b	0.414	down	-1.282	0.418	Down-regulated in breast[8], lung[9], ovarian[15], cervical[16], and prostate cancer[12]
mmu-miR-126-5p	0.424	down	-1.117	0.536	
hsa-miR-143	0.393	down	-1.245	0.605	Down-regulated in prostate[12], Lung[9], breast[8], hepatocellular[14], colon[7], cervical[16], and ovarian cancer[15]
hsa-miR-145	0.317	down	-2.130	0.899	Down-regulated in prostate[12], Lung[9], breast[8], hepatocellular[14], ovarian[15], cervical[16], and colon cancer[7]
hsa-miR-148b	0.317	down	-2.130	0.899	Down-regulated in pancreatic[17], and colon cancer[7]
hsa-miR-155	0.376	down	-1.374	0.486	Up-regulated in CLL[8], thyroid[10], lymphomas[13], lung[9], breast cancer[8] Down-regulated in pancreatic cancer[17]
hsa-miR-199a	0.261	down	-1.411	0.847	Down-regulated in prostate[12], and hepatocellular cancer[14]
hsa-miR-203	0.175	down	-1.925	0.910	Down-regulated in colon[7], and breast cancer[8] Up-regulated in ovarian cancer[15]

CLL: chronic lymphocytic leukemia

### Stem-loop qRT-PCR for miRNAs

**Figure 4:**
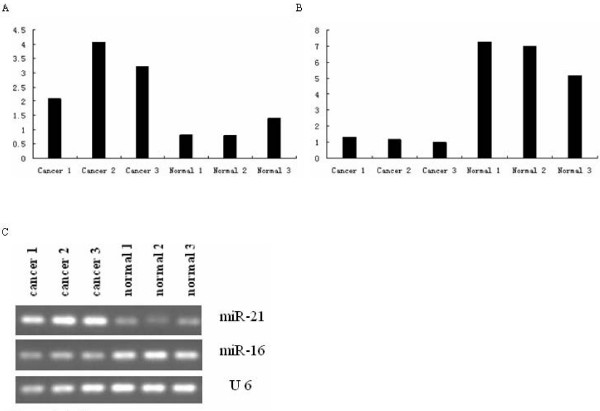
**qRT-PCR analysis of miR-21 and miR-16 in three cancer and three normal tissues**. A: The expression level of miR-21 in all samples. B: The expression level of miR-16 in all samples. C: The electrophoresis result of PCR products. U 6 expression was used as a loading control.

Our miRNA microarray detection platform was constructed by CapitalBio, and several previous comparative studies between microarray platforms and analysis procedures had indicated the very high sensitivity, reproducibility, and specificity using their recommended methods [28]. To confirm the microarray findings, we determined the hsa-miR-21 and hsa-miR-16 expression levels by Stem-loop qRT-PCR in all samples. The miRNAs were found to have the same expression levels as seen by microarray analysis (Figure 4).

## Discussion

Since Sally first established the DMBA-induced oral carcinogenesis model in the cheek pouch of Syrian hamster in 1954, it has become a classic animal model of OSCC [29]. In this study, we successfully constructed this animal model of OSCC using tri-weekly applications of a 5% solution of DMBA in acetone onto the cheek pouch of Syrian hamsters over about a 12-week period.

For models like the hamster model for OSCC, microarray assay provides a powerful tool for analyzing both miRNA expression patterns and quantitative expression levels, as it profiles thousands of genes simultaneously. This technology is much more efficient than the now outmoded and time-consuming methods used in earlier work, and is becoming the broadest miRNA research tool available [30]. We used a newly designed microarray platform specific for the analysis of the expression of some 924 mammalian miRNAs. The platform and assay are similar in many respects to other spotted oligonucleotide microarray designs, but have several important differences in application [24]. First, a modified spotting buffer and an advanced hybridization system were used in this study. These measures have both previously shown to result in large improvements in the local signal intensity and global signal uniformity, as well as in the elimination of the doughnut spots commonly seen on spotted oligonucleotide arrays. These improvements are believed to be due to better blocking of the slide surface chemistry [31]. A detailed assessment of the quality control and reproducibility of this new miRNA microarray platform has been published [32]. Using miRNA microarray analysis, we evaluated miRNA expression profiles of OSCC and normal cheek mucosa tissues, and identified seventeen miRNAs that were up-regulated and down-regulated in cancer tissues compared with normal tissues. In addition, hsa-miR-338, mmu-miR-762, and mmu-miR-126-5p were not apparently altered in any of the tumors.

Recently, there have been several studies regarding miRNA expression profiles of various tumor types and the general finding was that overall microRNA expression could differentiate normal versus cancerous tissues [7-17]. Among these previous studies, some miRNAs expression levels were similar to those found in the present study. These results are summarized in Table 2. Lu et al. has demonstrated the use of microRNA signatures as an important advance in cancer diagnosis. Their work indicated that microRNA-based identification of cancers was superior in terms of correctly diagnosing cancer of unknown primaries when compared to mRNA classification [33].

Hundreds of miRNAs have been identified in recent years and miRNA functional identification has become one of the most active research fields in biology. However, only a limited number of miRNAs has yet been defined functionally through overexpression, misexpression, and *in vitro *knockdown [34]. Recently, several studies have indicated that increased or decreased miRNA levels play a critical role in head and neck carcinogenesis. Using miRNA microarray analysis, Chang et al. identified seven miRNAs that were up-regulated (mir-21, let-7, 18, 29c, 142-3p, 155, and 146b) and one miRNA that was down-regulated (mir-494) in HNSCC primary tissue and cell lines. Moreover, they demonstrated that cytochrome c release was decreased by mir-21 knockdown, which suggested mir-21 inhibited several mRNAs that then led to a cascade of events that prevented apoptosis and increased cellular proliferation [35]. In addition, Tran et al. identified 54 commonly expressed miRNA genes, which included 31 up-regulated and 23 down-regulated miRNAs. The profiling data represented nine cell lines from four different anatomical head and neck sites [36]. In comparison to these previous studies, the expression tendency of four miRNAs (hsa-miR-21, hsa-miR-155, hsa-miR-200b, and hsa-miR-221) were found to be similar in our study. The similarity in expression of hsa-miR-21 in previous and our studies in head and neck squamous cell carcinoma and cancer cell lines is of particular interest. These findings, in conjunction with our study, demonstrate that miR-21 may play a critical role in head and neck carcinogenesis. This miRNA should therefore become a focus for the development of anti-microRNA preclinical therapeutic strategies for OSCC abrogation in the future.

Considering only the highly conserved microRNAs that were common in both humans and hamsters, we used the TargetScan program to check if the SAM-retrieved microRNAs were conservative types. In addition to mmu-miR-762 and mmu-miR-126-5p, fifteen other microRNAs were found highly conserved in most vertebrates. At present, mmu-miR-762 and mmu-miR-126-5p are not known to have been reported in any tumors.

Since miRNAs can function as oncogenes or as tumor suppressor genes, they provide a logical therapeutic target for cancer treatment [37]. Modified anti-miRNA oligonucleotides (AMOs) have been used by many groups to inhibit miRNAs with oncogenic properties. For example, Chan et al. successfully applied 2'-O-methyl- and DNA/LNA-mixed oligonucleotides to specifically knockdown miR-21, in order to investigate the potential contribution of this miRNA in the regulation of apoptosis-associated genes in glioblastoma cell lines [38]. Thus, to supplement and/or enhance the function of tumor suppressor miRNAs due to a deletion or a loss of function mutation, a therapeutic approach could entail exogenous delivery of corrective synthetic miRNAs in the form of double-stranded miRNA mimics [39]. Takamizawa et al. found that enforced expression of let-7 in the lung adenocarcinoma cell line A549 inhibited lung cancer cell growth *in vitro*. This holds promise that let-7 may be useful in treatment of lung cancer or in enhancing currently available treatments [40]. The microRNA field is rapidly developing, and the functions and signaling pathways of increasingly greater numbers of miRNAs are being carefully studied. The activation or silencing of miRNAs identified in the present study and in previous studies could prove pivotal in the design of therapeutic strategies for OSCC treatment in the future, although we are presently far from that point.

## Conclusion

In summary, the specific miRNA expression levels identified by our study were similar with those reported in other studies, and suggested that a number of miRNAs could be significant in OSCC development. The next step will be to perform functional research of the three microRNAs (hsa-miR-338, mmu-miR-762, and mmu-miR-126-5p) that were not found to have been altered in any malignancies.

## Abbreviations

DMBA: 7,12-dimethyl-benz [*a*]-anthrance; CLL: chronic lymphocytic leukemia; OSCC: oral squamous cell carcinoma; SAM: Significance Analysis of Microarrays.

## Competing interests

The authors declare that they have no competing interests.

## Authors' contributions

In our study, all authors have contributed significantly, and that all authors are in agreement with the content of the manuscript. Each author's contribution to the paper:TY: First author, background literature search, data analysis, development of final manuscript

XYW: Corresponding author, research instruction, data analysis, development of final manuscript. RGG: background literature search, data analysis. AL: research instruction, development of final manuscript. SY: research instruction, background literature search. YTC: data analysis, background literature search. YMW: research instruction, development of final manuscript. CMW: research instruction, data analysis. XZY: background literature search, data analysis.
